# Tracking of serum lipids from prepuberty to young adulthood: results from the KiGGS cohort study

**DOI:** 10.1186/s12944-024-02409-1

**Published:** 2024-12-26

**Authors:** Julia Truthmann, Anja Schienkiewitz, Antje Kneuer, Yong Du, Christa Scheidt-Nave

**Affiliations:** 1 Department of Epidemiology and Health Monitoring, Robert Koch-Institute Berlin, Berlin, Germany; 2 German Centre for Cardiovascular Research (DZHK), Partner Site Berlin, Berlin, Germany; 3 Department of General Practice, Institute for Community Medicine, University Medical Centre Greifswald, Greifswald, Germany

**Keywords:** Children, Young adults, Tracking, Serum lipids, Non-high-density lipoprotein, Prepuberty, Universal screening, Multifactorial dyslipidemia

## Abstract

**Background:**

Universal lipid screening in childhood for early detection and treatment of familial hypercholesterolemia is under discussion, but will also detect children with multifactorial dyslipidemia. Results from population-based studies can support the design of public health strategies. As few previous studies considered pubertal changes in serum lipid levels, we examined tracking of serum lipids from prepuberty to young adulthood in a population-based cohort.

**Methods:**

This longitudinal study includes 692 children from the German Health Interview and Examination Survey for Children and Adolescents (KiGGS; baseline: 2003–2006, follow-up: 2014–2017) who were 6–8 years old at baseline, at least 18 years old at follow-up, and had measurements of serum total cholesterol (TC), high-density and non-high-density lipoprotein cholesterol (HDL-C; non-HDL-C) at both time points. We calculated proportions of participants by life stage-specific risk categories applying cut points for young children and young adults. We used correlation coefficients to estimate serum lipid tracking from childhood to young adulthood. The association between follow-up and baseline lipid levels was examined in sex-specific multivariable linear regression models including body mass index (BMI), health-related behaviors and medication use as covariables.

**Results:**

The correlation coefficient between baseline and follow-up was 0.60 for non-HDL-C, 0.56 for TC, and 0.43 for HDL-C and was higher in males than in females. 67% of participants had acceptable and 9% had borderline/elevated non-HDL-C levels at both time points. Of participants with borderline/elevated non-HDL-C levels at baseline 32% remained in this category and 68% improved. Non-HDL-C levels at baseline explained 53% of the variance in levels at follow-up in males and 28% in females. After adjustment for covariables, the explained variance increased to 62% in males and 45% in females. An increase in BMI z-scores from childhood to young adulthood in all sexes and oral contraceptive use in females was positively associated with higher levels at follow-up.

**Conclusions:**

Non-HDL-C levels in prepuberty are moderate predictors of levels in young adulthood, along with increasing BMI from childhood to young adulthood, and oral contraceptive use among women. Comprehensive strategies including public health interventions targeting elevated lipid levels and obesity in combination, are essential to prevent premature cardiovascular events.

**Supplementary Information:**

The online version contains supplementary material available at 10.1186/s12944-024-02409-1.

## Background

Serum low-density lipoprotein cholesterol (LDL-C) as well as non-high-density lipoprotein cholesterol (non-HDL-C) levels in childhood and adolescence predict fatal and non-fatal atherosclerotic cardiovascular disease (ASCVD) in adulthood [1, 2]. There is strong evidence that children with heterozygous familial hypercholesterolemia (HeFH), which affects about 1 in 250 to 500 children and adolescents may benefit from early detection and lipid-lowering drug treatment, in order to prevent premature ACSVD [3, 4]. The benefits, risks and implications of universal cholesterol screening in childhood are nevertheless subject to ongoing debate [4–6]. As a much larger proportion of children with elevated lipid levels detected by screening will have multifactorial dyslipidemia requiring coordinated care and follow-up, public health strategies need to carefully designed [4].

Several previous population-based studies have analyzed the tracking of serum lipid levels from childhood to adulthood [7–22], and most have concluded that children with elevated serum lipid levels are likely to have elevated levels in (young) adulthood [8–19, 22, 23]. However, despite good correlation of lipid levels at both time points, up to 60% of children with elevated levels at baseline have lower levels at follow-up [8, 9, 14–16, 20]. Serum cholesterol concentrations undergo characteristic sex-specific changes during physical growth and sexual maturation [24–26]. In addition, serum lipid concentrations are apparently determined by other factors, including ethnicity [9, 14, 20, 22], poor intrauterine growth [27], oral contraceptive use [11–13, 28], body mass index, body composition [7–13, 22, 25], and behavioral factors, such as dietary habits [7], physical activity [7], alcohol consumption [7], smoking [7, 8].

Few previous population-based studies of serum lipid tracking from childhood to adulthood could exclude bias due to pubertal changes in serum lipid levels [10, 12–14, 20], and very few studies have studied tracking with respect to life stage-specific cut-off points recommended for clinical decision-making [19, 29]. Evidence is particularly scarce for non-HDL-C [30], although it may be at least as good a predictor of future ASCD as LDL-C and non-HDL measurement has the advantage of not being affected by fasting. The current study presents the longitudinal tracking of total cholesterol, non-HDL-C, and HDL-C levels from pre-puberty to young adulthood. In addition, we consider known modifiable determinants of elevated lipid levels at baseline and follow-up.

## Methods

### Study design

The German Health Interview and Examination Survey for Children and Adolescents (KiGGS) is part of the health monitoring framework in Germany and is designed to enable cross-sectional and longitudinal analyses [31]. The KiGGS baseline study was conducted from May 2003 to May 2006. The examination part of the follow-up survey KiGGS Wave 2 was carried out between September 2014 and August 2017. The design, sampling strategy and study protocol have been previously described in detail [31, 32]. In brief, participants were selected based on a two-staged sampling procedure. First 167 study locations (sample points) were selected proportional to the distribution of communities in Germany according to federal state, type of community, and population size. At the second stage within each sample point, children were sampled randomly from local population registries with stratification by sex and age. We obtained written consent from parents of all participating children irrespective of the child’s age and additionally from participants 14 years of age and older. The study was approved by the Ethics Committee at the Charité University Medicine Berlin, Germany.

### Study population

The population-based sample at baseline comprises 17641 participants aged 0–17 years living in Germany (response rate 67%). Of these, 6465 (37%) took part in the follow-up examinations.

**Fig. 1:**
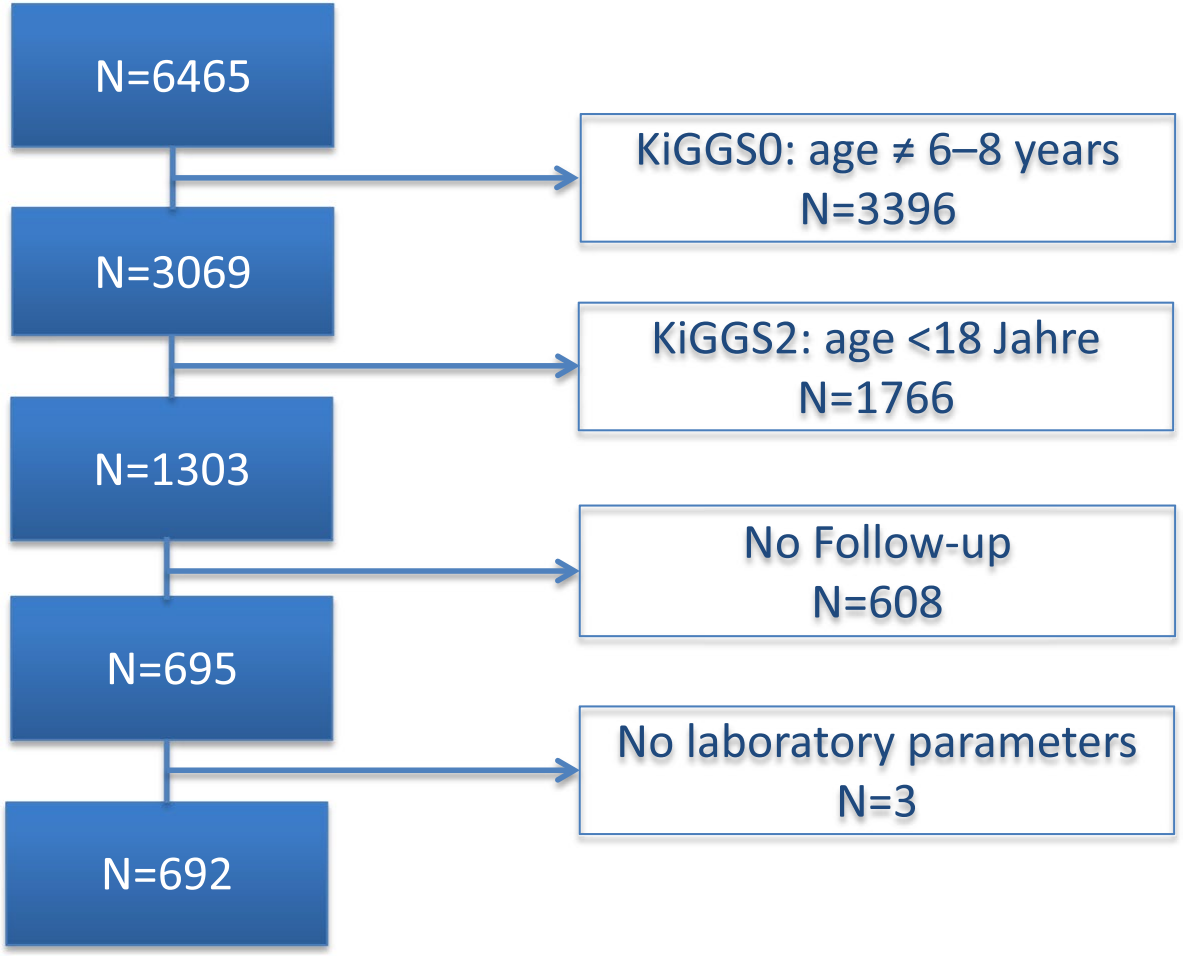
Flow diagram of study participants selection

Suggesting that the school entry health examination would be a practicable time point for universal screening the present analysis comprises children at the age of 6 – 8 years (considered as prepubertal) at baseline and at least 18 years at follow-up (considered as post-pubertal). Thus, the observed effects are not affected by pubertal changes in cholesterol levels. Participants with missing information on serum lipids at baseline or follow-up were excluded from the analysis (*N* = 611, Fig. 1). The present analysis is based on data of 692 children. Baseline characteristics of all KiGGS participants who were prepubertal at baseline and post-pubertal at follow-up are presented in Additional file 1 along with the weighted results for subsets of children with and without incomplete data. Mean serum lipids did not differ between all children potentially eligible for analysis and children in the final study population irrespective of considering survey-specific weighting or a two-component weighting factor additionally correcting for drop-out between baseline and follow-up. We observed no significant group differences with regard to sociodemographic characteristics, although children in the final study population were less likely to be female (51.2% vs 55.5%) and slightly older (8.01 vs 7.95 years).

### Serum lipid measurements

Venous blood samples were taken from children in a non-fasting state and blood specimens were processed within one hour according to a highly standardized protocol [33, 34]. Serum aliquots were frozen and transported on frozen cool packs at -40 °C to a central laboratory for analysis. At baseline serum lipid concentrations were analyzed using a cholesterol oxidase PAP method for TC and a homogeneous enzymatic color test for HDL-C (Hitachi 917, Roche, Mannheim, Germany) [35]. At follow-up, a cholesterol oxidase-based enzymatic color test was used to analyze TC concentrations and a homogeneous enzymatic color test with selective solvent was used to analyze HDL-C concentrations (Architect, Abbott, Wiesbaden, Germany). The principles of the analytical methods at baseline and follow-up were the same except that the manufacturers used different color reagents for the photometric detection for the quantitative determination of serum lipids. Results of comparative measurements with the analytical methods from baseline and follow-up showed a very good agreement of serum lipid concentrations (data not shown). Non-HDL-C was calculated as the difference between TC and HDL-C. Serum lipid levels were categorized (Table 1) according to the Expert Panel on Integrated Guidelines for Cardiovascular Health and Risk Reduction in Children and Adolescents [36]. While the diagnosis of HeFH requires clinical and genetic testing, serum levels of total cholesterol exceeding 6.98 mmol/l (270 mg/dL) are considered indicative of possible HeFH [4].

**Table 1:** Serum lipid level categories according to the NHLBI expert panel [36]

		**Acceptable**	**Borderline**	**Elevated**
**Non-HDL-C**	Children	<3.11 mmol/l < 120 mg/dl	3.11–3.75 mmol/l 120–144 mg/dl	≥ 3.76 mmol/l ≥ 145 mg/dl
	Young Adults	<3.89 mmol/l < 150 mg/dl	3.89–4.91 mmol/l 150–189 mg/dl	≥ 4.92 mmol/l ≥ 190 mg/dl
**TC**	Children	< 4.40 mmol/l < 170 mg/dl	4.40–5.17 mmol/l 170–199 mg/dl	≥ 5.18 mmol/l ≥ 200 mg/dl
	Young Adults	<4.92 mmol/l < 190 mg/dl	4.92–5.82 mmol/l 190–224 mg/dl	≥ 5.83 mmol ≥ 225 mg/dl
		**Acceptable**	**Borderline**	**Low**
**HDL-C**	Children and Young Adults	> 1.17 mmol/l > 45 mg/dl	1.04–1.17 mmol/l 40–45 mg/dl	< 1.04 mmol/l < 40 mg/dl

*NHLBI* National Heart, Lung and Blood Institute

### Covariables

The following risk factors are known as potential determinants of unfavorable lipid levels: a diet rich in saturated fat and low in fiber, increased body weight, physical inactivity, a risky alcohol intake, smoking [37] and oral contraceptive use [28]. The Healthy Food Diversity index (HFD) was calculated based on 38 food items among children and 44 food items among young adults. The index considers three aspects: the number (n), distribution, and health value of all consumed foods and is bounded between 0 and 1–1/n. Higher HFD values reflect a healthier diet. Detailed information on index construction are provided elsewhere [29]. Body height and body weight were measured by trained staff. Body mass index (BMI) z-scores were calculated [38] and obesity classified according to the national reference system [39, 40]. Among children, physical activity within and outside sports clubs was determined based on following categories: “never”, “1–2 times a month”, “1–2 times a week”, “3–5 times a week”, and “every day” [41] and classified as low (< 1 time a week), middle (1–2 times a week) and high (≥ 3 times a week). Information on physical activity among young adults was obtained based on self-report using standardized self-administered questionnaires. Participants were asked: “How much time do you spent on sports, fitness or physical activity in an ordinary week?”. Extreme values above the 95th percentile were winsorized (replaced by 95th percentile). Alcohol consumption among young adults was assessed with four questions [42]. One question on lifetime prevalence of alcohol consumption (response categories: “yes” or “no”) and three questions of the brief alcohol screen AUDIT-C (Alcohol Use Disorders Identification Test-Consumption). The three questions lead to a final score between 0 and 12 points. Alcohol consumption was categorized as “no”, “moderate” (< 4 points among females; < 5 points among males) and “at-risk” (≥ 4 points among females; ≥ 5 points among males). Information on current cigarette smoking was assessed with questionnaires. Any medication use in last seven days was recorded in a standardized interview (AMEDA). Classification was based on the most currently available version of the Anatomical Therapeutic Chemical (ATC) classification system. ATC codes were used to define oral contraceptive use (G03A). The collection of study variables was described in detail elsewhere [32, 34].

### Statistical analysis

Analyses were conducted using SAS 9.4 (SAS Institute, Cary, NC). *P*-values < 0.05 were considered statistically significant. All analyses were weighted using a special weighting factor to account for potential bias due to two components: drop-out between baseline and follow-up and selective participation (correction for deviations from the population structure). Logistic regression models were used to calculate the population weight based on logistic regression models, taking into account the deviation between the study sample and the population structure (31st December 2004) according to age, sex, region, parental education and nationality. To consider the cluster design of the sample and the correlation of the participants within a community, the confidence intervals are determined with the survey procedures for complex samples of SAS 9.4. Percentages, means and 95%-confidence intervals (95%-CI) were calculated. Pearson’s correlation coefficients of continuous serum lipid levels were calculated. Furthermore, the proportion of participants with acceptable and borderline/elevated levels at both time points was calculated. Linear regression models stratified by sex were used to analyze the bivariable associations between serum non-HDL-C, TC, HDL-C at follow-up and the respective childhood baseline serum lipid levels as well as the following non-lipid determinants: BMI z-score at baseline and difference from baseline to follow-up, food diversity index at baseline and difference from baseline to follow-up, physical activity at baseline and sports at follow-up, smoking at follow-up, alcohol use at follow-up and oral contraceptive use at follow-up (among female participants). In addition, multivariable models were fitted. Sensitivity analyses were performed for non-HDL-C as the outcome variable in two steps. First, we repeated analyses excluding individuals with TC levels > 6.98 mmol/l as well as persons with a diagnosis of diabetes or on medications potentially affecting serum lipid levels including lipid-lowering drugs and systemic corticosteroids (sensitivity analyses 1). Sensitivity analyses 2 additionally excluded individuals taking oral contraceptives.

## Results

Characteristics of the study population are presented in Table 2. The mean age at baseline was 8.0 years and the proportion of girls was 51.2%. At baseline 6.0% of individuals had obesity, no child had diabetes or took lipid-lowering medications, systemic corticosteroids, or oral contraceptives. Mean follow-up time of the cohort was 10.9 years. From school entry age at baseline to young adulthood at follow-up mean serum non-HDL-C increased from 2.79 to 3.03 mmol/l, mean TC increased from 4.33 to 4.43 mmol/l, and mean HDL-C decreased from 1.54 to 1.40 mmol/l. At baseline 72.0% of the cohort had acceptable non-HDL-C levels, 20.7% had borderline non-HDL-C levels and 7.2% exceeded the currently recommended cut-point of 3.76 mmol/l (144 mg/dL). One individual at baseline had TC levels higher than 6.98 mmol/l (270 mg/dl) indicating possible familial hypercholesterolemia. This amounts to an estimated prevalence of 0.4%; Table 2). At follow-up six individuals exceeded the cut-point of 6.98 mmol/l (1.1%).

**Table 2:** Study population characteristics; individuals aged 6–8 years at baseline and ≥ 18 years at follow-up (*N* = 692)

% or Mean (95%-CI)	Baseline (2003–2006)	Follow-up (2014–2017)
	***N* = 692 persons**	***N* = 692 persons**
Sex (% female)	51.2 (46.3–56.1)	51.2 (46.3–56.1)
Age (years)	8.0 (7.9–8.1)	18.9 (18.8–19.0)
**Serum lipids**		
Non-HDL-C (mmol/l)	2.79 (2.72–2.87)	3.03 (2.93–3.13)
TC (mmol/l)	4.33 (4.26–4.41)	4.43 (4.33–4.53)
HDL-C (mmol/l)	1.54 (1.51–1.57)	1.40 (1.37–1.43)
**Serum lipid categories (%)**		
Non-HDL-C		
acceptable	72.0 (67.4–76.2)	61.4 (56.5–66.1)
borderline	20.7 (17.1–24.8)	22.8 (18.9–27.3)
high	7.2 (5.0–10.5)	15.7 (12.3–19.9)
TC		
acceptable	55.6 (50.9–60.2)	53.7 (48.5–58.7)
borderline	34.5 (30.2–39.0)	26.1 (22.0–30.7)
high	9.9 (7.2–13.4)	20.2 (16.7–24.3)
HDL-C (mmol/l)		
acceptable	87.7 (83.8–90.7)	76.0 (71.1–80.3)
borderline	8.0 (5.5–11.5)	13.3 (10.4–16.8)
low	4.3 (2.7–6.9)	10.8 (7.8–14.8)
TC > 270mg/dl/6.98mmol/l (familial hypercholesterolemia)	0.4 (0.1–3.1)	1.1 (0.3–3.7)
**Obesity and behavioral determinants**		
BMI z-score	0.06 (-0.05–0.17)	0.05 (-0.08–0.19)
Obesity (%)	6.0 (4.0–8.9)	6.9 (4.7–10.0)
Diabetes mellitus (%)	0	0.3 (0.1–1.2)
HFD index score	0.55 (0.54–0.57)	0.36 (0.35–0.38)
Physical activity per week (%)		n. a
Low	17.7 (13.7–22.6)	
Middle	36.2 (31.3–41.4)	
High	46.1 (41.1–51.1)	
Sports (h/week)	n. a	3.4 (3.1–3.7)
Smoking (%)	n. a	30.8 (26.3–25.7)
Alcohol use (%)	n. a	
No		19.5 (15.1–24.9)
Moderate		32.6 (27.8–37.8)
At risk		47.9 (43.0–52.9)
**Medication use (%)**		
Oral contraceptives (among women, *N* = 343)	0	26.6 (23.0–30.7)
Lipid lowering-medication use	0	0.4 (0.1–3.0)
Systemic corticosteroids	0	0.1 (0.0–0.8)

Baseline: Baseline: KiGGS baseline (2003–2006), Follow-up: KiGGS wave 2 (2014–2017)

Cut-points for serum lipids for children and young adults according to Expert Panel on Integrated Guidelines for Cardiovascular Health and Risk Reduction in Children and Adolescents [36], see Table 1

*HDL-C* High density lipoprotein cholesterol, *HFD* Healthy Food Diversity index, *non-HDL-C* Non-High-density lipoprotein cholesterol, *KIGGS* German Health Interview and Examination Survey for Children and Adolescents, *TC* Total cholesterol

*N* Missing Baseline: BMI = 2, HFD index = 24, physical activity = 40, diabetes = 7

*N* Missing Follow-up: BMI = 3, HFD index = 18, sports = 20, smoking = 15, alcohol use = 16, oral contraceptive use = 6, lipid-lowering medication use = 6, systemic corticosteroids = 6, diabetes = 2

*N* fulfilling exclusion criteria for sensitivity analyses (at baseline): TC > 270mg/dl = 1

*N* fulfilling exclusion criteria for sensitivity analyses (at follow-up): TC > 270mg/dl = 6, diabetes = 2, oral contraceptive use = 193, lipid-lowering medication = 1, systemic corticosteroids = 1

**Fig. 2:**
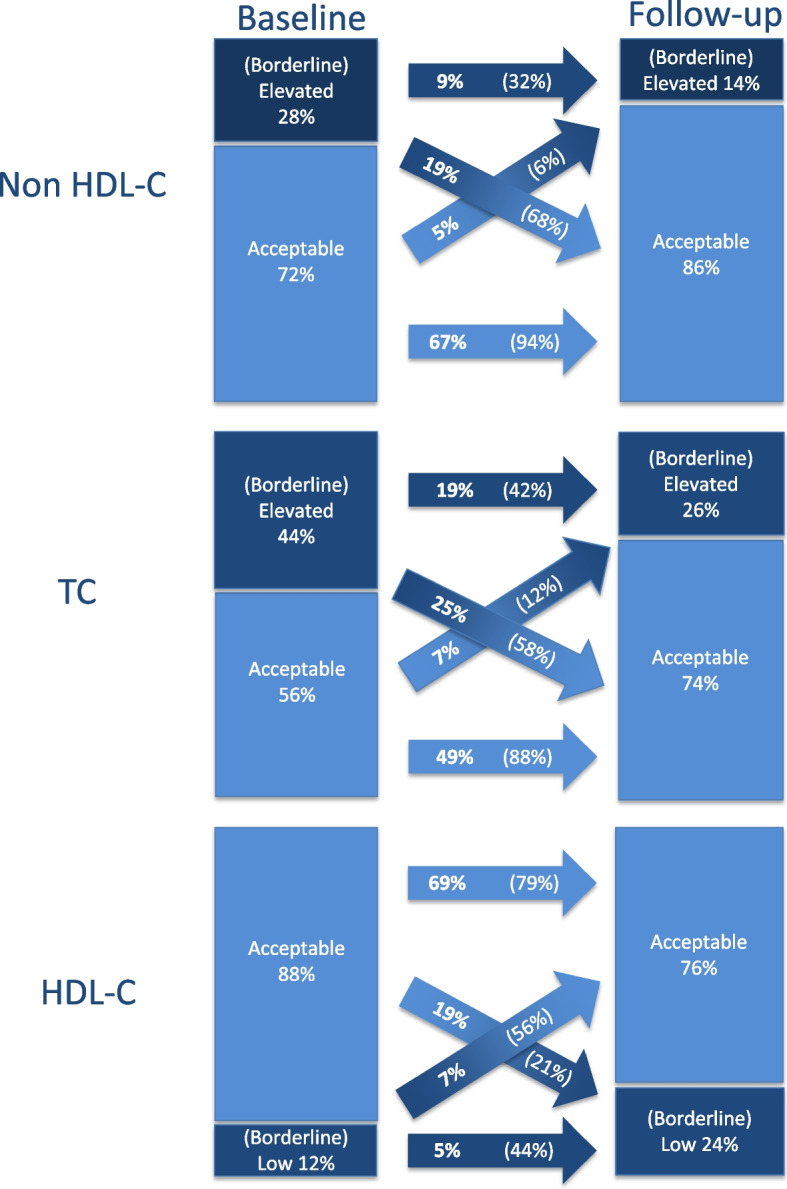
Change in serum lipid levels from childhood to young adulthood (*N* = 692). HDL-C: high density lipoprotein cholesterol, non-HDL-C: Non-High-density lipoprotein cholesterol, TC: total cholesterol. Percent change in categories from baseline to follow-up is depicted in the arrows. For proportions in bold print the numerator is the whole study cohort; for proportions in parentheses the numerator is the total number of individuals in that particular baseline risk category. Cut-offs for serum lipids according to Expert Panel on Integrated Guidelines for Cardiovascular Health and Risk Reduction in Children and Adolescents [36], see Table 1

Changes in risk categories of non-HDL-C, TC, and HDL-C from childhood to young adulthood are presented in Fig. 2. With regard to non-HDL-C, a total of 76% of the study cohort persisted in their initial category as compared to 68% for TC and 74% for HDL-C. Among cohort members with borderline/elevated level at baseline 32% (CI 24–41%) remained in that category and 68% (CI 59–76%) improved their non-HDL-C to acceptable levels at follow-up in young adulthood (remission). Sensitivity analysis excluding individuals with TC > 6.98 mmol/l at baseline or follow-up as well as young adults with diabetes or medication use affecting serum lipid levels (lipid lowering drugs; systemic corticosteroids) showed similar results (Additional file 3, Figure S1). In sensitivity analyses also excluding women on oral contraceptives at follow-up, there was a lower persistence in the borderline/elevated category from baseline to follow-up (see Figure S2, 20% instead of 32%). Table 3 presents the change in risk categories for the subgroup of children with elevated Non-HDL-C levels at baseline (*N* = 53). 24% (CI 10-46%) of children with elevated non-HDL-C levels persisted in this category, 29% (CI 18-44%) had borderline levels and 47% (CI 29-67%) had acceptable levels at follow-up.

**Table 3:** Serum lipid risk categories in adulthood among children aged 6–8 years^b^ with elevated serum levels

	**Lipid risk category at follow-up**^**a**^	*N*	% (95%-CI)
Baseline non-HDL-C ≥ 3.76 mmol/l^a^ (*N* = 53)	Acceptable Non-HDL-C	25	47.1 (29.0-66.0)
	Borderline Non-HDL-C	20	29.2 (17.7-44.0)
	Elevated Non-HDL-C	8	23.7 (10.1-46.2)
Baseline TC ≥ 5.18 mmol/l^a^ (*N* = 70)	Acceptable TC	21	26.6 (16.4-40.2)
	Borderline TC	25	29.2 (17.3-45.0)
	Elevated TC	24	44.1 (28.3-61.3)
Baseline HDL-C < 1.04 mmol/l^a^ (*N* = 29)	Acceptable HDL-C	16	54.5 (31.0-76.2)
	Borderline HDL-C	7	27.1 (10.6-53.8)
	Low HDL-C	6	18.4 (5.5-46.9)

^a^Cut-points for serum lipids according to Expert Panel on Integrated Guidelines for Cardiovascular Health and Risk Reduction in Children and Adolescents [36], see Table 1. The percentages shown have been rounded to one decimal place, resulting in a deviation of 0.1% from 100%

^b^*N* = 692 persons 6–8 years of age at KiGGS baseline and ≥ 18 + years at KiGGS wave 2

For TC, persistence in the baseline category was 68% (Fig. 2). 42% (36-49%) of children with borderline/high TC levels at baseline remained at the same level at follow-up. 44% (CI 28-61%) of children with elevated TC at baseline (*N* = 70) remained in their initial category (see Table 3).

Persistence of HDL-C levels in the initial category was 74%. 44% (30-58%) of children with borderline/low HDL-C levels at baseline remained in the borderline/low category at follow-up (Fig. 2). 18% (6-47%) with low HDL-C at baseline (N=29) remained in the initial category (see Table 3).

Correlation coefficients for baseline and follow-up serum lipid levels were 0.60 for non-HDL-C, 0.56 for TC, and 0.43 for HDL-C and generally higher among male (non-HDL-C: r = 0.67, TC: r = 0.63, HDL-C: r = 0.60) than female cohort members (non-HDL-C: r = 0.53, TC: r = 0.51, HDL-C: r = 0.48). Sensitivity analysis for non-HDL-C showed similar results (sensitivity analysis 1: male r = 0.61, female r = 0.53; sensitivity analysis 2: male r = 0.61, female r = 0.58;).

Table 4 presents the association of follow-up levels of non-HDL-C and its determinants. Among male participants, non-HDL-C levels at baseline explained 53% of variance of non-HDL-C at follow-up (beta = 0.95). After adjustment for covariables explained variance increased to 62%. An increase in BMI z-score from childhood to young adulthood (beta = 0.28) was associated with higher non-HDL-C levels among males. Among female participants baseline levels of non-HDL-C explained 28% of variance of follow-up levels (beta = 0.67). Oral contraceptive use in young adulthood (beta = 0.67) and an increase in BMI z-score from childhood to young adulthood (beta = 0.17) were associated with higher non-HDL-C levels at follow-up.

**Table 4:** Determinants of non-high-density lipoprotein cholesterol in young adulthood (*N* = 594 persons^a^)

	**Male (*N* = 306)**				**Female (*N* = 288)**			
	**bivariable**		**multivariable**		**bivariable**		**multivariable**	
	**Beta (95% CI)**	**R**^**2**^	**Beta (95% CI)**	**R**^**2**^	**Beta (95% CI)**	**R**^**2**^	**Beta (95% CI)**	**R**^**2**^
**Baseline non-HDL cholesterol**	0.95 (0.68;1.23)***	0.53	0.81 (-0.18;1.37)***	0.62	0.67 (0.50–0.83)***	0.28	0.67 (0.54;0.79)***	0.45
BMI z-score in childhood	0.18 (-0.04;0.41)	0.04	0.06 (-0.01;0.13)		0.05 (-0.06;0.16)	< 0.01	-0.01 (-0.10;0.08)	
Difference in BMI z-score to young adulthood	0.40 (0.18–0.63)***	0.17	0.28 (0.17;0.39)***		0.14 (-0.03;0.30)	0.02	0.17 (0.08;0.25)***	
Smoking in young adulthood	-0.18 (-0.50;0.14)	< 0.01	-0.08 (-0.23; 0.08)		0.21 (-0.15;0.58)	0.01	0.20 (-0.08;0.49)	
Alcohol use in young adulthood		0.07				< 0.01		
No	0.74 (-0.14;1.63)		0.40 (-0.02; 0.82)		-0.09 (-0.25;0.33)		0.24 (-0.14;0.61)	
Moderate	ref		ref		ref		ref	
At risk	-0.06 (-0.29;0.17)		-0.01 (-0.15;0.14)		0.06 (-0.25;0.36)		-0.08 (-0.28; 0.12)	
Oral contraceptives use in young adulthood	-	-	-	-	0.68 (0.44;0.92)***	0.16	0.67 (0.47;0.87)***	

The regression model includes BMI z-score in childhood and difference in BMI z-score in young adulthood, healthy food diversity index in childhood and change in young adulthood, physical activity in childhood, sports in young adulthood, smoking in young adulthood, alcohol use in young adulthood and oral contraceptive use in young adulthood. Variables explaining less than 1% of the variance in both sexes are not shown in the tables of the main manuscript. The full model is shown in Additional file 2

^**a**^6–8 years of age at KiGGS baseline and ≥ 18 + years at KiGGS wave 2

^*^p < 0.05, **p < 0.01, ***p < 0.001

The results of the regression models for TC (Table 5) were similar to the results for non-HDL-cholesterol. Explained variance of follow-up levels of TC by baseline levels were 53% among males and 30% among females. Explained variance in the multivariable models were 62% among males (46% among females) after adjustment for additional determinants of follow-up levels. Oral contraceptive use (among females) and an increase in BMI z-score from childhood to young adulthood (among all sexes) were associated with higher TC levels at follow-up.

**Table 5:** Determinants of total cholesterol in young adulthood (*N* = 594 persons^a^)

	**Male (*N* = 306)**				**Female (*N* = 288)**			
	**bivariable**		**multivariable**		**bivariable**		**multivariable**	
	**Beta (95% CI)**	**R**^**2**^	**Beta (95% CI)**	**R**^**2**^	**Beta (95% CI)**	**R**^**2**^	**Beta (95% CI)**	**R**^**2**^
**Baseline total cholesterol**	0.95 (0.70;1.20)***	0.53	0.85 (0.70;1.00)***	0.62	0.75 (0.54;0.96)***	0.30	0.71 (0.54; 0.87)***	0.46
BMI z-score in childhood	0.16 (-0.06; 0.38)	0.03	0.08 (0.00;0.15)		0.05 (-0.09;0.18)	< 0.01	0.03 (-0.07; 0.14)	
Difference in BMI z-score to young adulthood	0.36 (0.14; 0.57)***	0.13	0.25 (0.15; 0.35)***		0.10 (-0.10;0.31)	< 0.01	0.14 (0.04; 0.24)**	
Alcohol use in young adulthood		0.06				< 0.01		
No	0.64 (-0.18; 1.46)		0.44 (0.09; 0.78)		-0.11 (-0.64; 0.42)		0.23 (-0.18; 0.64)	
Moderate	ref		ref		ref		ref	
At risk	-0.07 (-0.31; 0.16)		0.05 (-0.09; 0.19)		0.02 (-0.33; 0.37)		-0.09 (-0.32; 0.14)	
Oral contraceptives use in young adulthood	-	-	-	-	0.85 (0.56; 1.13)***	0.20	0.81 (0.57; 1.04)***	

The regression model includes BMI z-score in childhood and difference in BMI z-score in young adulthood, healthy food diversity index in childhood and change in young adulthood, physical activity in childhood, sports in young adulthood, smoking in young adulthood, alcohol use in young adulthood and oral contraceptive use in young adulthood. Variables explaining less than 1% of the variance in both sexes are not shown in the tables of the main manuscript. The full model is shown in Additional file 2

^**a**^6–8 years of age at KiGGS baseline and ≥ 18 + years at KiGGS wave 2

^*^p < 0.05, **p < 0.01, ***p < 0.001

Table 6 presents regression models for HDL-C follow-up levels/. Explained variance for follow-up levels by baseline levels were 32% among males and 19% among females. In multivariable models explained variance was 37% for males and 27% for females. Oral contraceptive use (among females) were associated with higher HDL-C levels at follow-up.

**Table 6:** Determinants of high-density lipoprotein cholesterol in young adulthood (*N* = 594 persons^a^)

	**Male (*N* = 306)**				**Female (*N* = 288)**			
	**bivariable**		**multivariable**		**bivariable**		**multivariable**	
	**Beta (95% CI)**	**R**^**2**^	**Beta (95% CI)**	**R**^**2**^	**Beta (95% CI)**	**R**^**2**^	**Beta (95% CI)**	**R**^**2**^
**Baseline HDL cholesterol**	0.39 (0.31; 0.48)***	0.32	0.38 (0.29; 0.47)***	0.37	0.47 (0.30; 0.63)***	0.19	0.46 (0.28; 0.63)***	0.27
HFD index in childhood	0.26 (0.06; 0.46)*	0.02	0.20 (-0.06; 0.46)		0.11 (-0.26; 0.49)	< 0.01	0.21 (-0.19; 0.61)	
HFD index in young adulthood	-0.16 (-0.37; 0.05)	< 0.01	0.00 (-0.22; 0.21)		0.17 (-0.11; 0.44)	< 0.01	0.27 (-0.06; 0.59)	
Physical activity in childhood		0.01				< 0.01		
Low	-0.08 (-0.19; 0.03)		-0.01 (-0.10; 0.07)		-0.02 (-0.17; 0.13)		0.02 (-0.11;0.14)	
Middle	-0.06 (-0.12; 0.00)		-0.05 (-0.11; 0.01)		0.04 (-0.05; 0.14)		0.05 (-0.04; 0.13)	
High	ref		ref		ref		ref	
Sports (h per week) in young adulthood	0.01 (0.00;0.02)*	0.02	0.00 (-0.01; 0.01)		0.00 (-0.02; 0.01)	< 0.01	-0.01 (-0.02; 0.01)	
Oral contraceptives use in young adulthood	-	-	-	-	0.17 (0.07; 0.26)***	0.06	0.16 (0.07; 0.24)***	

The regression model includes BMI z-score in childhood and difference in BMI z-score in young adulthood, healthy food diversity index in childhood and change in young adulthood, physical activity in childhood, sports in young adulthood, smoking in young adulthood, alcohol use in young adulthood and oral contraceptive use in young adulthood. Variables explaining less than 1% of the variance in both sexes are not shown in the tables of the main manuscript. The full model is shown in Additional file 2

^a^6–8 years of age at KiGGS baseline and ≥ 18 + years at KiGGS wave 2

^*^p < 0.05, **p < 0.01, ***p < 0.001

Sensitivity analyses conducted for non-HDL-C as the outcome variable excluding individuals with TC levels > 6.98 mmol/l, diabetes mellitus or use of medications affecting serum lipids (lipid-lowering medication, oral corticosteroids) were consistent with the results presented in Table 4 (see Table S5 in Additional File 3). In the sensitivity analyses that additionally excluded females using oral contraceptives (see Table S6), the explained variance in the multivariable model was 48% in men and 52% in women. In both females and males, baseline non-HDL-C levels and an increase in BMI z-score from childhood to young adulthood were associated with higher levels at follow-up.

## Discussion

### Main results and comparison to previous studies

In the present study non-HDL-C levels in childhood were moderate predictors of levels in young adulthood, with an explained variance of 41% among males and 28% among females. The correlation coefficient between baseline and follow-up TC levels was 0.56, with slightly higher correlations among males (0.63) compared to females (0.51), which is in line with previous studies [10, 12, 14]. The Cardiovascular Risk in Young Finns study [10] found that TC baseline levels in girls aged 3–9 years were the major predictor of follow-up levels at the age of 15–21 years, explaining 22% of the variance (39% in males). Similar results were observed in the Bogalusa Heart study [14], where baseline TC levels of 2–8 year-old children were the strongest predictor of follow-up levels at the age of 20–26 years. In the Muscatine study baseline TC levels of 7–8 year-old girls explained 42% (boys: 31%) of the levels at the age of 20–25 years [12].

The present study showed an increase of mean serum non-HDL-C and mean TC levels and a decrease of mean HDL-C levels from school entry age at baseline to young adulthood at follow-up. These findings can be expected based on results from cross-sectional and longitudinal studies demonstrating fluctuations during natural growth and maturation from childhood to adulthood [24, 26]. In addition to baseline serum lipid levels, which were the strongest predictor of follow-up levels, oral contraceptive use (in young women) and an increase in z-BMI from childhood to young adulthood were potential risk factors for elevated levels of non-HDL-C and TC levels in young adulthood. This finding is consistent with those of previous studies, which have demonstrated an association of BMI [10, 12, 14] and oral contraceptive use [12] with serum lipid levels in young adulthood. As shown in a previous analysis of KiGGS baseline data [28], teenage girls using contraceptives had a more unfavorable cardiometabolic profile compared to non-users. There was also a clustering of behavioral risk factors in association with oral contraceptive use including current smoking, regular alcohol consumption and lack of sports activity [28]. In addition to the studies investigating the correlation of lipid levels at two time points the i3C Consortium (International Childhood Cardiovascular Cohorts) also examined the association between childhood non-HDL-C levels and atherosclerotic cardiovascular disease risk over a 35-year follow-up period, finding that higher non–HDL-C levels in childhood were associated with a higher risk of fatal events even after adjusting for other factors [2]. A different analysis of the data of the i3C Consortium showed that LDL-C levels were consistently significantly higher among children who had a higher BMI z-score [43].

This previous population-based study found a prevalence of 7.2% children with multifactorial dyslipidemia measured by non-HDL-C level at the age of 6–8 years. Only one child at the age of 6–8 years had TC levels higher than 6.98 mmol/l indicating familial hypercholesterolemia. The first results of the Fridolin Trial [44], which comprises children at the age of 2–6 years in Lower Saxony (Germany) indicate a prevalence of LDL-hypercholesterolemia of 2.2%. Comparing these data is difficult due to the different measurement methods, definitions of dyslipidemia and the different age groups. In three US population-based studies the prevalence of FH ranged from 0.2% to 0.4% [45], which is comparable to our findings.

The association between baseline and follow-up lipid levels was consistently stronger in males than in females. The difference may be partly associated to oral contraceptive use in young women, as the effect was attenuated after taking oral contraceptive use into account. Sensitivity analysis 2 excluding individuals with TC > 6.98 mmol/l or diabetes or medication (oral contraceptives, systemic corticosteroids or lipid-lowering) showed comparable values of explained variance for females and males. This finding emphasizes the association between oral contraceptive use and serum lipid levels, as well as a clustering of behavioral cardiovascular risk factors identified in a previous study [28]. However, since the model excluded half of the females in our analysis, the observed association may be partially attributable to overfitting.

### Strengths and weaknesses

The major strengths of the present study are the tracking of serum lipids in a nationwide, population-based sample of prepubertal children with a follow-up period of 11 years. However, some limitations have to be acknowledged. Firstly, due to the longitudinal dropout of the study group there might be a selection bias resulting in a higher proportion of health-conscious participants. To correct for the longitudinal dropout all analyses were weighted using a study specific weighting factor and the distribution of study characteristics showed good agreement between the entire KiGGS subset eligible for analysis and the final study population Secondly, serum lipid levels have a large intra-individual variation [20]. Since analyses in this study were based on a single measurement the association might be over- or underestimated. Thirdly, the present analysis focused on potentially modifiable determinants in school age children or young adults. We did not consider pregnancy or birth history, in particular fetal growth retardation which has been shown to adversely affect serum lipid levels [27]. Objective information on family predisposition to hypercholesterinemia based on genetic parameters was not assessed. A previous study identified genetic risk scores that may add to the prediction of dyslipidemia [46] and therefore inclusion of this score may improve the identification of children at risk in lipid screening programs. Another limitation which should be mentioned is the assessment of health behavior with self-administered questionnaires. This has the potential for reporting bias and might have resulted in residual confounding. Furthermore, the FFQ is primarily designed to rank persons according to their diet not to assess absolute intake of single meal components like dietary fat.

### Implications and future research

The present study analyzed correlation of serum lipid levels in childhood and young adulthood and the tracking of categorized serum lipid levels. Previous studies have shown that serum lipid levels increase with age until puberty, decline thereafter and increase again in young adulthood [24, 43, 47]. In addition, female individuals generally have higher lipid levels than male individuals at each stage of puberty. The success of a universal screening program relies on the sensitivity and specificity of the screening cut-offs. A false-positive result can lead to uncertainty and helplessness of the people affected. False-negative screening results may provide a false sense of security. Therefore, age- and sex-specific cut-offs are needed to account for fluctuations in lipid levels during growth. Population-specific reference values should be considered in the development of future guidelines for dyslipidemia in children and adolescents. This will improve the identification of those at risk who need clinical assessment and further evaluation of cardiovascular risk factors.

Screening for multifactorial dyslipidemia will identify individuals who should receive lifestyle interventions and medical treatment. The association between increasing BMI levels between childhood and young adulthood and higher non-HDL levels in young adulthood. Both obesity and multifactorial dyslipidemia share underlying risk factors such as poor diet, physical inactivity, sedentary behavior including excessive screen time, and environmental risk factors e.g. marketing of unhealthy foods, limited access to healthy foods and environments that do not encourage physical activity. Evidence suggests that public health strategies targeting the broader societal and environmental factors are more effective than individual lifestyle interventions [48]. A comprehensive approach is needed to reduce the prevalence of obesity as well as the prevalence of multifactorial dyslipidemia, thereby reducing the economic and individual burden of the disease.

The finding that a large proportion of the variance in lipid levels in young adulthood in female individuals remained unexplained by modifiable determinants and its implications for screening for lipid levels in childhood require further investigation. Girls tend to have higher levels of TC and low-density lipoprotein cholesterol than boys in childhood, but these levels may change during puberty and adulthood [49]. In adult women, LDL-C levels are generally lower while HDL-C levels are higher than in men, especially in early adulthood. Puberty and associated hormonal changes play a critical role in altering lipid profiles.

## Conclusion

Prepubertal non-HDL-C levels serve as moderate predictors of non-HDL-C levels in young adulthood, particularly in male individuals. In addition to childhood lipid levels, obesity in male individuals and oral contraceptive use in female individuals are associated with elevated non-HDL-C levels in young adulthood. A large proportion of the variance of lipid levels in young adulthood among female individuals remained unexplained by modifiable determinants, which requires further investigation. The findings in male individuals highlight the importance of public health interventions targeting lipid levels and obesity at the population level to reduce the prevalence of multifactorial dyslipidemia. A comprehensive strategy of public health interventions, identification of individuals at risk and subsequent individualized lifestyle interventions or medical treatment is essential to prevent premature cardiovascular events [21].

## Supplementary Information

Additional file 1: Baseline characteristics for participants with complete and incomplete data. The Additional File 1 presents the baseline characteristics for participants with complete and incomplete baseline and follow-up information.

Additional file 2: Additional file 2 shows the full regression models including modifiable risk factors that explain less than 1% of the variance.

Additional file 3: The Additional File 3 shows sensitivity analyses excluding individuals with TC levels > 6.98 mmol/l, diabetes, or medication use.

## Data Availability

The authors confirm that some access restrictions apply to the data underlying the findings. The data set cannot be made publicly available because informed consent from study participants did not cover public deposition of data. However, the minimal data set underlying the findings is archived in the 'Health Monitoring' Research Data Centre at the Robert Koch Institute (RKI) and can be accessed by all interested researchers. On-site access to the data set is possible at the Secure Data Center of the RKI's 'Health Monitoring' Research Data Centre. Requests should be submitted to the 'Health Monitoring' Research Data Centre, Robert Koch Institute, Berlin, Germany (e-mail: fdz@rki.de).

## Abbreviations

ASCVD: Atherosclerotic cardiovascular disease
ATC: Anatomical Therapeutic Chemical
AUDIT-C: Alcohol Use Disorders Identification Test-Consumption
BMI: Body mass index
HeFH: Heterozygous familial hypercholesterolemia
HFD: Healthy Food Diversity index
HDL-C: High density lipoprotein cholesterol
KiGGS: German Health Interview and Examination Survey for Children and Adolescents
LDL-C: Low-density lipoprotein cholesterol
non-HDL-C: non-high-density lipoprotein cholesterol
TC: Total cholesterol
