# Supplementary material for: Tracking of serum lipids from prepuberty to young adulthood: results from the KiGGS cohort study
Source: Lipids Health Dis. 2024 Dec 26;23:421. doi: 10.1186/s12944-024-02409-1 (PMC11670486; doi:10.1186/s12944-024-02409-1)
Supplement: Supplementary file 1 — Additional file 1: Baseline characteristics for participants with complete and incomplete data. The Additional File 1 presents the baseline characteristics for participants with complete and incomplete baseline and follow-up information. [file 12944_2024_2409_MOESM1_ESM.docx]

**Additional File 1**

**Table S1: Comparison of baseline characteristics between children with and without complete data**

|  | **All participants aged 6-8 years at baseline and >=18 years at follow-up*** | **Participants excluded^#^*** | **Participants with baseline and follow- up data*** | **Participants with baseline and follow- up data**** |
| --- | --- | --- | --- | --- |
| **% or Mean (95%-CI)** | **N=1303** | **N=611** | **N=692** | **N=692** |
|  |  |  |  |  |
| Sex (% girls) | 55.5 (52.7-58.2) | 59.7 (55.5-63.7) | 51.7 (47.3-56.0) | 51.2 (46.3-56.1) |
| Age | 7.95 (7.91-7.99) | 7.93 (7.87-7.99) | 7.96 (7.91-8.02) | 8.01 (7.95-8.09) |
| Parental  educational status |  |  |  |  |
| Low | 25.2 (21.5-29.4) | 29.2 (23.7-35.3) | 21.7 (17.4-26.5) | 27.5 (22.1-33.5) |
| Middle | 49.3 (45.2-53.3) | 45.9 (40.3-51.7) | 52.2 (47.6-56.8) | 48.5 (43.7-53.4) |
| High | 25.5 (22.6-28.7) | 24.9 (21.2-29.0) | 26.1 (22.5-30.1) | 24.0 (20.5-28.0) |
| Serum lipids |  |  |  |  |
| Non-HDL-C (mmol/l) | 2.81 (2.76-2.85) | 2.80 (2.73-2.87) | 2.81 (2.75-2.87) | 2.79 (2.72-2.87) |
| TC (mmol/l) | 4.34 (4.30-4.39) | 4.34 (4.26-4.42) | 4.35 (4.29-4.41) | 4.33 (4.26-4.41) |
| HDL-C (mmol/l) | 1.54 (1.51-1.57) | 1.54 (1.50-1.58) | 1.54 (1.51-1.57) | 1.54 (1.51-1.57) |

# no follow-up or exclusion because of missing values
*weighted using a weighting factor to correct deviations from the population structure in Germany with regard to age, sex, region and nationality as of 31.12.2004
** weighted using a two-component weighting factor to correct for drop-out (between baseline and follow-up) and to correct for deviations from the population structure in Germany with regard to age, sex, region, and nationality as of December 31^st^, 2004
