# Supplementary material for: Tracking of serum lipids from prepuberty to young adulthood: results from the KiGGS cohort study
Source: Lipids Health Dis. 2024 Dec 26;23:421. doi: 10.1186/s12944-024-02409-1 (PMC11670486; doi:10.1186/s12944-024-02409-1)
Supplement: Supplementary file 2 — Additional file 2: Additional file 2 shows the full regression models including modifiable risk factors that explain less than 1% of the variance. [file 12944_2024_2409_MOESM2_ESM.docx]

**Table S2:** **Determinants of non-high-density lipoprotein cholesterol in young adulthood (****N=594 persons^#^)**

|  | **Male (N=306)** | | | | | | **Female (N=288)** | | | | |
| --- | --- | --- | --- | --- | --- | --- | --- | --- | --- | --- | --- |
|  | **bivariable** | | | **multivariable** | | | **bivariable** | | **multivariable** | | |
|  | **Beta (95% CI)** | **R²** | **Beta (95% CI)** | | **R²** | **Beta (95% CI)** | | **R²** | **Beta (95% CI)** | **R²** |  |
| **Baseline non-HDL cholesterol** | 0.95 (0.68;1.23)*** | 0.53 | 0.81 (-0.18;1.37)*** | | 0.62 | 0.67 (0.50-0.83)*** | | 0.28 | 0.67 (0.54;0.79)*** | 0.45 |  |
| BMI z-score in childhood | 0.18 (-0.04;0.41) | 0.04 | 0.06 (-0.01;0.13) | |  | 0.05 (-0.06;0.16) | | <0.01 | -0.01 (-0.10;0.08) |  |  |
| Difference in BMI z-score to young adulthood | 0.40 (0.18-0.63)*** | 0.17 | 0.28 (0.17;0.39)*** | |  | 0.14 (-0.03;0.30) | | 0.02 | 0.17 (0.08;0.25)*** |  |  |
| HFD index in childhood | 0.16 (-0.66;0.98) | <0.01 | -0.02 (-0.93; 0.90) | |  | -0.04 (-0.94;0.86) | | <0.01 | -0.28 (-1.29;0.73) |  |  |
| HFD index in young adulthood | -0.09 (-1.24;1.07) | <0.01 | 0.00 (-0.81; 0.82) | |  | 0.00 (-0.74;0.73) | | <0.01 | -0.23 (-1.21;0.75) |  |  |
| Physical activity in childhood |  | <0.01 |  | |  |  | | <0.01 |  |  |  |
| Low | 0.06 (-0.20;0.31) |  | -0.12 (-0.35; 0.11) | |  | -0.18 (-0.54;0.19) | |  | 0.12 (-0.07;0.31) |  |  |
| Middle | 0.17 (-0.24;0.58) |  | 0.11 (-0.10;0.32) | |  | 0.08 (-0.19;0.35) | |  | 0.03 (-0.19;0.25) |  |  |
| High | ref |  | ref | |  | ref | |  | ref |  |  |
| Sports (h per week) in young adulthood | 0.01 (-0.05;0.07) | <0.01 | -0.01 (-0.03;0.02) | |  | -0.01 (-0.01; 0.04) | | <0.01 | -0.02 (-0.05;0.00) |  |  |
| Smoking in young adulthood | -0.18 (-0.50;0.14) | <0.01 | -0.08 (-0.23; 0.08) | |  | 0.21 (-0.15;0.58) | | 0.01 | 0.20 (-0.08;0.49) |  |  |
| Alcohol use in young adulthood |  | 0.07 |  | |  |  | | <0.01 |  |  |  |
| No | 0.74 (-0.14;1.63) |  | 0.40 (-0.02; 0.82) | |  | -0.09 (-0.25;0.33) | |  | 0.24 (-0.14;0.61) |  |  |
| Moderate | ref |  | ref | |  | ref | |  | ref |  |  |
| At risk | -0.06 (-0.29;0.17) |  | -0.01 (-0.15;0.14) | |  | 0.06 (-0.25;0.36) | |  | -0.08 (-0.28; 0.12) |  |  |
| Oral contraceptives use in young adulthood | - | - | - | | - | 0.68 (0.44;0.92)*** | | 0.16 | 0.67 (0.47;0.87)*** |  |  |

**^#^**6-8 years of age at KiGGS baseline and ≥18+ years at KiGGS wave 2
*p<0.05, **p<0.01, ***p<0.001
HFD: Healthy Food Diversity index

**Table S3: Determinants of total cholesterol in young adulthood (N=594 persons^#^)**

|  | **Male (N=306)** | | | | | | **Female (N=288)** | | | | |
| --- | --- | --- | --- | --- | --- | --- | --- | --- | --- | --- | --- |
|  | **bivariable** | | | **multivariable** | | | **bivariable** | | **multivariable** | | |
|  | **Beta (95% CI)** | **R²** | **Beta (95% CI)** | | **R²** | **Beta (95% CI)** | | **R²** | **Beta (95% CI)** | **R²** |  |
| **Baseline total cholesterol** | 0.95 (0.70;1.20)*** | 0.53 | 0.85 (0.70;1.00)*** | | 0.62 | 0.75 (0.54;0.96)*** | | 0.30 | 0.71 (0.54; 0.87)*** | 0.46 |  |
| BMI z-score in childhood | 0.16 (-0.06; 0.38) | 0.03 | 0.08 (0.00;0.15) | |  | 0.05 (-0.09;0.18) | | <0.01 | 0.03 (-0.07; 0.14) |  |  |
| Difference in BMI z-score to young adulthood | 0.36 (0.14; 0.57)*** | 0.13 | 0.25 (0.15; 0.35)*** | |  | 0.10 (-0.10;0.31) | | <0.01 | 0.14 (0.04; 0.24)** |  |  |
| HFD index in childhood | 0.42 (-0.43;1.28) | <0.01 | 0.23 (-0.59;1.06) | |  | 0.08 (-1.04;1.20) | | <0.01 | -0.14 (-1.07; 0.79) |  |  |
| HFD index in young adulthood | -0.24 (-1.39; 0.89) | <0.01 | 0.07 (-0.75; 0.88)) | |  | 0.17 (-0.68;1.02) | | <0.01 | 0.03 (-0.91; 1.00) |  |  |
| Physical activity in childhood |  | <0.01 |  | |  |  | | <0.01 |  |  |  |
| Low | -0.02 (-0.33;0.29) |  | -0.10 (-0.33; 0.12) | |  | -0.20 (-0.63; 0.23) | |  | 0.14 (-0.07; 0.34) |  |  |
| Middle | 0.11 (-0.29;0.51) |  | 0.06 (-0.13; 0.24) | |  | 0.12 (-0.17; 0.42) | |  | 0.08 (-0.15; 0.31) |  |  |
| High | ref |  | ref | |  | ref | |  | ref |  |  |
| Sports (h per week) in young adulthood | 0.02 (-0.04;0.08) | <0.01 | -0.01 (-0.04; 0.12) | |  | -0.01 (-0.06; 0.04) | | <0.01 | -0.03 (-0.06; 0.00) |  |  |
| Smoking in young adulthood | -0.19 (-0.51; 0.13) | <0.01 | -0.12 (-0.27; 0.03) | |  | 0.15 (-0.26; 0.56) | | <0.01 | 0.10 (-0.18; 0.39) |  |  |
| Alcohol use in young adulthood |  | 0.06 |  | |  |  | | <0.01 |  |  |  |
| No | 0.64 (-0.18; 1.46) |  | 0.44 (0.09; 0.78) | |  | -0.11 (-0.64; 0.42) | |  | 0.23 (-0.18; 0.64) |  |  |
| Moderate | ref |  | ref | |  | ref | |  | ref |  |  |
| At risk | -0.07 (-0.31; 0.16) |  | 0.05 (-0.09; 0.19) | |  | 0.02 (-0.33; 0.37) | |  | -0.09 (-0.32; 0.14) |  |  |
| Oral contraceptives use in young adulthood | - | - | - | | - | 0.85 (0.56; 1.13)*** | | 0.20 | 0.81 (0.57; 1.04)*** |  |  |

**^#^**6-8 years of age at KiGGS baseline and ≥18+ years at KiGGS wave 2
*p<0.05, **p<0.01, ***p<0.001
HFD: Healthy Food Diversity index

**Table S4: Determinants of high-density lipoprotein cholesterol in young adulthood (N=594 persons^#^)**

|  | **Male (N=306)** | | | | | | **Female (N=288)** | | | | |
| --- | --- | --- | --- | --- | --- | --- | --- | --- | --- | --- | --- |
|  | **bivariable** | | | **multivariable** | | | **bivariable** | | **multivariable** | | |
|  | **Beta (95% CI)** | **R²** | **Beta (95% CI)** | | **R²** | **Beta (95% CI)** | | **R²** | **Beta (95% CI)** | **R²** |  |
| **Baseline HDL cholesterol** | 0.39 (0.31; 0.48)*** | 0.32 | 0.38 (0.29; 0.47)*** | | 0.37 | 0.47 (0.30; 0.63)*** | | 0.19 | 0.46 (0.28; 0.63)*** | 0.27 |  |
| BMI z-score in childhood | -0.02 (-0.05; 0.01) | <0.01 | 0.00 (-0.02; 0.02) | |  | -0.01 (-0.06; 0.05) | | <0.01 | 0.03 (-0.01; 0.08) |  |  |
| Difference in BMI z-score to young adulthood | -0.05 (-0.08; -0.02) | <0.01 | -0.04 (-0.07; -0.02) | |  | -0.04 (-0.10; 0.02) | | <0.01 | -0.04 (-0.08; 0.01) |  |  |
| HFD index in childhood | 0.26 (0.06; 0.46)* | 0.02 | 0.20 (-0.06; 0.46) | |  | 0.11 (-0.26; 0.49) | | <0.01 | 0.21 (-0.19; 0.61) |  |  |
| HFD index in young adulthood | -0.16 (-0.37; 0.05) | <0.01 | 0.00 (-0.22; 0.21) | |  | 0.17 (-0.11; 0.44) | | <0.01 | 0.27 (-0.06; 0.59) |  |  |
| Physical activity in childhood |  | 0.01 |  | |  |  | | <0.01 |  |  |  |
| Low | -0.08 (-0.19; 0.03) |  | -0.01 (-0.10; 0.07) | |  | -0.02 (-0.17; 0.13) | |  | 0.02 (-0.11;0.14) |  |  |
| Middle | -0.06 (-0.12; 0.00) |  | -0.05 (-0.11; 0.01) | |  | 0.04 (-0.05; 0.14) | |  | 0.05 (-0.04; 0.13) |  |  |
| High | ref |  | ref | |  | ref | |  | ref |  |  |
| Sports (h per week) in young adulthood | 0.01 (0.00;0.02)* | 0.02 | 0.00 (-0.01; 0.01) | |  | 0.00 (-0.02; 0.01) | | <0.01 | -0.01 (-0.02; 0.01) |  |  |
| Smoking in young adulthood | -0.01 (-0.07; 0.05) | <0.01 | -0.03 (-0.09; 0.03) | |  | -0.06 (-0.016; 0.04) | | <0.01 | -0.09 (-0.19; 0.00) |  |  |
| Alcohol use in young adulthood |  | <0.01 |  | |  |  | | <0.01 |  |  |  |
| No | -0.10 (-0.20; 0.00) |  | -0.02 (-0.13; 0.09) | |  | -0.02 (-0.19; 0.15) | |  | 0.00 (-0.13; 0.13) |  |  |
| Moderate | ref |  | ref | |  | ref | |  | ref |  |  |
| At risk | -0.02 (-0.07; 0.04) |  | 0.02 (-0.04; 0.07) | |  | -0.04 (-0.16; 0.09) | |  | -0.02 (-0.12; 0.09) |  |  |
| Oral contraceptives use in young adulthood | - | - | - | | - | 0.17 (0.07; 0.26)*** | | 0.06 | 0.16 (0.07; 0.24)*** |  |  |

**^#^**6-8 years of age at KiGGS baseline and ≥18+ years at KiGGS wave 2
*p<0.05, **p<0.01, ***p<0.001
HFD: Healthy Food Diversity index
