# Supplementary material for: Tracking of serum lipids from prepuberty to young adulthood: results from the KiGGS cohort study
Source: Lipids Health Dis. 2024 Dec 26;23:421. doi: 10.1186/s12944-024-02409-1 (PMC11670486; doi:10.1186/s12944-024-02409-1)
Supplement: Supplementary file 3 — Additional file 3: The Additional File 3 shows sensitivity analyses excluding individuals with TC levels > 6.98 mmol/l, diabetes, or medication use. [file 12944_2024_2409_MOESM3_ESM.docx]

**Sensitivity analyses 1:**

**
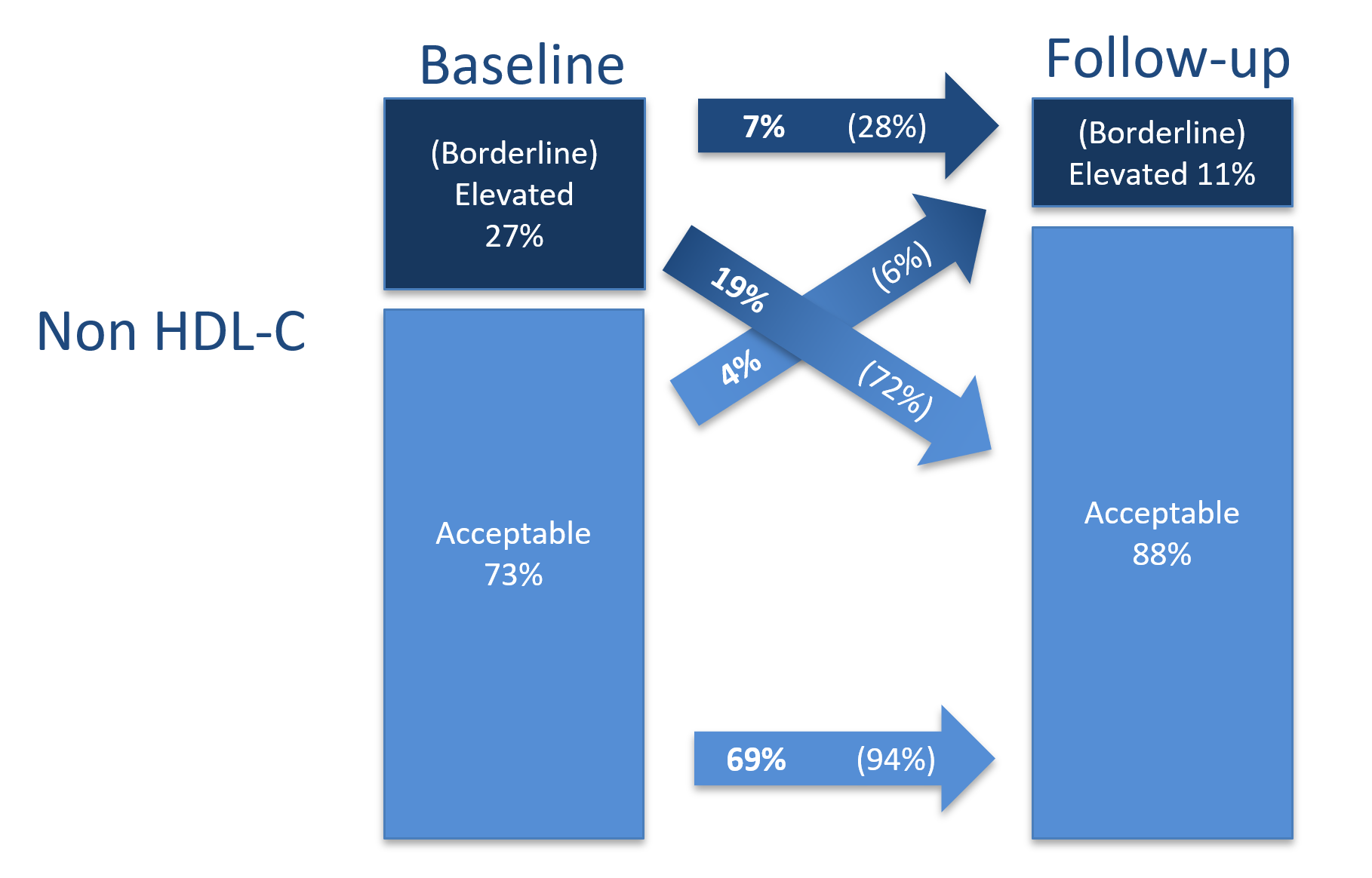
**

**Figure S1: Change in non-high-density lipoprotein cholesterol levels from childhood to young adulthood (Sensitivity analysis 1, N=681). Analyses excluding individuals with TC levels>6.98mmol/l, diabetes mellitus, and medication use with effect on serum lipid levels (lipid-lowering drugs, systemic corticosteroids).**

Percent change in risk categories from baseline to follow-up is depicted in the arrows. For proportions in bold print the numerator is the whole study cohort; for proportions in parentheses the numerator is the total number of individuals in that particular baseline risk category.
Cut-offs for serum lipids according to Expert Panel on Integrated Guidelines for Cardiovascular Health and Risk Reduction in Children and Adolescents (36):
Borderline/elevated non-HDL-C: baseline (≥3.11 mmol/l), follow up (≥3.89 mmol/l)

**Table S5: Determinants of non-high-density lipoprotein cholesterol in young adulthood, N=585 persons 6-8 years of age at KiGGS baseline and ≥18+ years at KiGGS wave 2 (Sensitivity analysis 1, N=585). Analyses excluding individuals with TC levels>6.98mmol/l, diabetes mellitus, and medication use with effect on serum lipid levels (lipid-lowering drugs, systemic corticosteroids).**

|  | **Male (N=303)** | | | | | | **Female (N=282)** | | | | |
| --- | --- | --- | --- | --- | --- | --- | --- | --- | --- | --- | --- |
|  | **bivariable** | | | **multivariable** | | | **bivariable** | | **multivariable** | | |
|  | **Beta (95% CI)** | **R²** | **Beta (95% CI)** | | **R²** | **Beta (95% CI)** | | **R²** | **Beta (95% CI)** | **R²** |  |
| **Baseline level** | 0.79 (0.69;0.89)*** | 0.40 | 0.73 (0.63;0.82)*** | | 0.48 | 0.62 (0.47;0.78)*** | | 0.30 | 0.61 (0.50;0.72)*** | 0.48 |  |
| BMI z-score in childhood | 0.03 (-0.07;0.14) | <0.01 | 0.02 (-0.03;0.08) | |  | 0.10 (-0.02;0.21) | | 0.01 | 0.02 (-0.06;0.11) |  |  |
| Difference in BMI z-score to young adulthood | 0.29 (0.16;0.41)*** | 0.12 | 0.24 (0.15;0.33)*** | |  | 0.09 (-0.05;0.24) | | 0.01 | 0.13 (0.05;0.20)** |  |  |
| HFD index in childhood | 0.28 (-0.42;0.96) | <0.01 | -0.19 (-0.98;0.60) | |  | 0.23 (-0.58;1.04) | | <0.01 | 0.26 (-0.47;0.99) |  |  |
| Difference in HFD index score to young adulthood | -0.44 (-1.12;0.24) | 0.01 | -0.28 (-0.99;0.42) | |  | 0.22 (-0.46;0.90) | | <0.01 | 0.21 (-0.53;0.94) |  |  |
| Physical activity in childhood |  | <0.01 |  | |  |  | | <0.01 |  |  |  |
| Low | 0.04 (-0.22;0.29) |  | -0.10 (-0.30;0.11) | |  | -0.10 (-0.44;0.25) | |  | 0.18 (0.02;0.35)* |  |  |
| Middle | -0.08 (-0.30;0.14) |  | 0.02 (-0.13;0.16) | |  | 0.11 (-0.13;0.34) | |  | 0.08 (-0.10;0.26) |  |  |
| High | ref |  | ref | |  | ref | |  | ref |  |  |
| Sports (h per week) in young adulthood | -0.01 (-0.04;0.03) | <0.01 | -0.02 (-0.04;0.01) | |  | 0.00 (-0.04;0.04) | | <0.01 | -0.02 (-0.04;0.01) |  |  |
| Smoking in young adulthood | -0.18 (-0.38;0.02) | 0.01 | -0.07 (-0.23;0.08) | |  | 0.26 (-0.08;0.60) | | 0.02 | 0.26 (0.00;0.51) |  |  |
| Alcohol use in young adulthood |  | 0.02 |  | |  |  | | <0.01 |  |  |  |
| No | 0.28 (-0.12;0.67) |  | 0.17 (-0.07;0.41) | |  | -0.16 (-0.57;0.25) | |  | 0.20 (-0.15;0.54) |  |  |
| Moderate | ref |  | ref | |  | ref | |  | ref |  |  |
| At risk | -0.11 (-0.31;0.10) |  | -0.03 (-0.17;0.10) | |  | -0.01 (-0.30;0.27) | |  | -0.14 (-0.33;0.05) |  |  |
| Oral contraceptives use in young adulthood | - |  |  | |  | 0.62 (0.41;0.84)*** | | 0.17 | 0.62 (0.45;0.79)*** |  |  |

*p<0.05, **p<0.01, ***p<0.001
HFD: Healthy Food Diversity index

**Sensitivity analyses 2:**


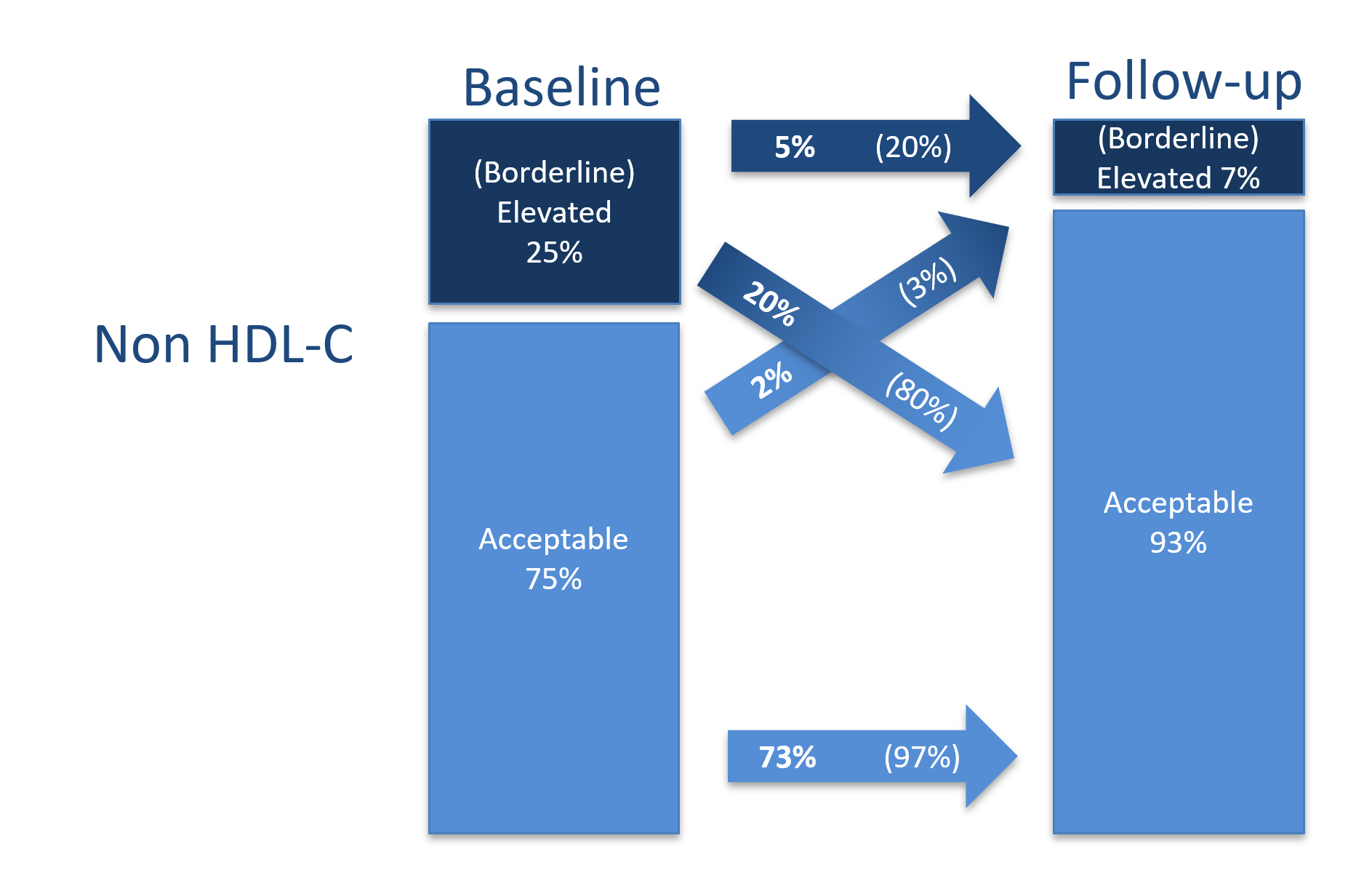


**Figure S2: Change in non-high-density lipoprotein cholesterol levels from childhood to young adulthood (Sensitivity analysis 2, N=493). Analyses excluding individuals with TC levels>6.98mmol/l, diabetes mellitus, and medication use with effect on serum lipid levels (lipid-lowering drugs, systemic corticosteroids, oral contraceptives).**

Percent change in categories from baseline to follow-up is depicted in the arrows. For proportions in bold print the numerator is the whole study cohort; for proportions in parentheses the numerator is the total number of individuals in that particular baseline risk category. Cut-offs for serum lipids according to Expert Panel on Integrated Guidelines for Cardiovascular Health and Risk Reduction in Children and Adolescents 36:
Borderline/elevated non-HDL-C: baseline (≥3.11 mmol/l), follow up (≥3.89 mmol/l)

**Table S6: Association between follow-up levels of non-high-density lipoprotein cholesterol and determinants of non-high-density lipoprotein cholesterol in young adulthood, N=420 persons 6-8 years of age at KiGGS baseline and ≥18+ years at KiGGS wave 2 (Sensitivity analysis 2, N=420). Analyses excluding individuals with TC levels>6.98mmol/l, diabetes mellitus, and medication use with effect on serum lipid levels (lipid-lowering drugs, systemic corticosteroids, oral contraceptives).**

|  | **Male (N=303)** | | | | | | **Female (N=117)** | | | | |
| --- | --- | --- | --- | --- | --- | --- | --- | --- | --- | --- | --- |
|  | **bivariable** | | | **multivariable** | | | **bivariable** | | **multivariable** | | |
|  | **Beta (95% CI)** | **R²** | **Beta (95% CI)** | | **R²** | **Beta (95% CI)** | | **R²** | **Beta (95% CI)** | **R²** |  |
| **Baseline level** | 0.79 (0.69;0.89)*** | 0.40 | 0.73 (0.63;0.82)*** | | 0.48 | 0.54 (0.39;0.69)*** | | 0.32 | 0.59 (0.45;0.73)*** | 0.51 |  |
| BMI z-score in childhood | 0.03 (-0.08;0.14) | <0.01 | 0.02 (-0.03;0.08) | |  | 0.12 (-0.02;0.27) | | 0.03 | 0.03 (-0.06;0.12) |  |  |
| Difference in BMI z-score to young adulthood | 0.29 (0.16;0.41)*** | 0.13 | 0.24 (0.15;0.33)*** | |  | 0.12 (-0.02;0.27) | | 0.03 | 0.14 (0.05;0.23)** |  |  |
| HFD index in childhood | 0.27 (-0.41;0.96) | <0.01 | -0.19 (-0.98;0.60) | |  | 0.46 (-0.34;1.27) | | <0.01 | 0.63 (-0.37;1.63) |  |  |
| Difference in HFD index score to young adulthood | -0.44 (-1.11;0.24) | 0.01 | -0.28 (-0.99;0.43) | |  | 0.34 (-0.37;1.05) | | <0.01 | 0.53 (-0.37;1.43) |  |  |
| Physical activity in childhood |  | <0.01 |  | |  |  | | 0.01 |  |  |  |
| Low | 0.04 (-0.22;0.29) |  | -0.10 (-0.30;0.11) | |  | 0.05 (-0.30;0.40) | |  | 0.29 (0.09;0.50)* |  |  |
| Middle | -0.08 (-0.30;0.14) |  | 0.02 (-0.13;0.16) | |  | 0.26 (-0.16;0.68) | |  | 0.27 (0.00;0.53) |  |  |
| High | ref |  | ref | |  | ref | |  | ref |  |  |
| Sports (h per week) in young adulthood | -0.01 (-0.04;0.03) | <0.01 | -0.02 (-0.04;0.00) | |  | -0.05 (-0.10;0.00) | | 0.04 | -0.05 (-0.08;-0.02)* |  |  |
| Smoking in young adulthood | -0.18 (-0.38;0.02) | 0.01 | -0.07 (-0.23;0.08) | |  | 0.11 (-0.46;0.68) | | <0.01 | 0.45 (0.04;0.86) |  |  |
| Alcohol use in young adulthood |  | 0.02 |  | |  |  | | <0.01 |  |  |  |
| No | 0.28 (-0.12;0.67) |  | 0.17 (-0.07;0.41) | |  | 0.10 (-0.34;0.55) | |  | 0.20 (-0.15;0.54) |  |  |
| Moderate | ref |  | ref | |  | ref | |  | ref |  |  |
| At risk | -0.11 (-0.31;0.10) |  | -0.03 (-0.17;0.10) | |  | -0.05 (-0.37;0.27) | |  | -0.19 (-0.41;0.04) |  |  |

*p<0.05, **p<0.01, ***p<0.001
HFD: Healthy Food Diversity index
